# DAMPs Synergize with Cytokines or Fibronectin Fragment on Inducing Chondrolysis but Lose Effect When Acting Alone

**DOI:** 10.1155/2017/2642549

**Published:** 2017-07-18

**Authors:** Lei Ding, Joseph A. Buckwalter, James A. Martin

**Affiliations:** ^1^Department of Basic Medical Sciences, Jiangnan University Wuxi Medical School, Wuxi, Jiangsu, China; ^2^Department of Orthopaedics and Rehabilitation, University of Iowa Hospitals and Clinics, Iowa City, IA, USA; ^3^Veterans Affairs Medical Center, Iowa City, IA, USA

## Abstract

**Objective and Design:**

To investigate whether endogenous damage-associated molecular patterns (DAMPs) or alarmins originated from mitochondria or nucleus stimulates inflammatory response in articular chondrocytes to cause chondrolysis which leads to cartilage degradation featured in posttraumatic osteoarthritis (PTOA).

**Materials:**

Primary cultures of bovine or human chondrocytes isolated from cartilage of weight-bearing joints.

**Treatment:**

Chondrocytes were subjected to mitochondrial DAMPs (MTDs) or HMGB1, a nuclear DAMP (NuD), with or without the presence of an N-terminal 29 kDa fibronectin fragment (Fn-f) or proinflammatory cytokines (IL-1*β* and TNF-*α*). Injured cartilage-conditioned culturing medium containing a mixture of DAMPs was employed as a control. After 24 hrs, the protein expression of cartilage degrading metalloproteinases and iNOS in culture medium or cell lysates was examined with Western blotting, respectively.

**Results:**

HMGB1 was synergized with IL-1*β* in upregulating expression of MMP-3, MMP-13, ADAMTS-5, ADAM-8, and iNOS. Moreover, a moderate synergistic effect was detected between HMGB1 and Fn-f or between MTDs and TNF-*α* on MMP-3 expression. However, when acting alone, MTDs or HMGB1 did not upregulate cartilage degrading enzymes or iNOS.

**Conclusion:**

MTDs or HMGB1 could only stimulate inflammatory response in chondrocytes with the presence of cytokines or Fn-f.

## 1. Introduction

Joint injury frequently causes progressive degradation of cartilage leading to joint pain, stiffness, and loss of motility which are the clinical manifestations of posttraumatic osteoarthritis (PTOA) [1–5]. How joint trauma induces progressive and irreversible degradation of articular cartilage remains poorly understood. As an avascular and nerveless tissue, cartilage responds to mechanical insults differently from vascularized tissues. Chondrocytes, the only cell type in cartilage, are responsible for trauma-induced degradation of the collagen and proteoglycan-rich extracellular matrix (ECM). Understanding of molecular pathways that lead to cartilage destruction will help to develop strategies that have the potential to prevent injured joints from having PTOA.

Chondrocyte death is one of the important biological events immediately following joint trauma. When an injury occurs to a weight-bearing joint, articular surface sustains an injurious blow from the sudden loss of joint stability. Several studies employing ex vivo models to mimic this type of impaction on cartilage revealed that significant amount of chondrocyte death quickly following the injury was observed in the superficial tangential zone of the tissue from which this phenomenon then turned into a slower propagating “wave of cell death” with time [6–11]. This impaction-induced chondrocyte death was later confirmed in in vivo studies that reported more profound effect compared to ex vivo studies, which showed complete loss of cells spanning the full thickness of injured cartilage only weeks after the insult [12–14].

The direct consequence of chondrocyte death is the release of intracellular contents; some of which are capable of priming immune cells including dendritic cells, T cells, and macrophages to trigger inflammation [15–17]. Collectively, in response to “danger” or “damage,” those immune activators derived endogenously from stressed or injured tissues are structurally distinct and become rapidly available in peripheral tissues. Based on this nature, they are termed as “alarmins.” Together with pathogen-associated molecular patterns (PAMPs), alarmins and PAMPs form a group termed danger/damage-associated molecular patterns (DAMPs) and alarmins are also termed as endogenous DAMPs [18–20].

A recent study indicated that mitochondrial DAMPs (MTDs), contents released from ruptured mitochondria caused by mechanical trauma, were capable of stimulating migration and degranulation of polymorphonuclear neutrophils (PMNs) to trigger innate immunity leading to neutrophil-mediated organ injury usually observed in a systemic inflammatory response syndrome (SIRS) [21]. In addition to MTDs, HMGB1, a nucleus-originated DAMP (NuD), was demonstrated as another systemic inflammation mediator in a murine fracture model. The study showed that HMGB1 signaled through membrane toll-like receptor 4 (TLR4) located on the apical surface of intestinal cells to elicit inflammatory response and end-organ injury following bilateral femur fracture [22].

However, it still remains unclear how chondrocytes respond to MTDs or HMGB1 released from ruptured mitochondria or nuclei following joint trauma. Would they evoke catabolic reactions in chondrocytes as other proinflammaotry mediators, like inflammatory cytokines [23] or fibronectin fragments (Fn-fs) [24, 25]? Would synergism exist among them? To address these questions, we tested the effect of MTDs or HMGB1 either in singular or in combinations with inflammatory cytokines or Fn-fs on the upregulation of catabolic metalloproteinases (MMPs) or iNOS in chondrocyte cultures.

## 2. Materials and Methods

### 2.1. Acquisition and Culturing of Bovine and Human Articular Chondrocytes

Full-thickness bovine cartilage shaved from stifle joints was subjected to 0.4% protease (Sigma-Aldrich®, St. Louis, MO) and then to 0.02% collagenase (Sigma-Aldrich, St. Louis, MO) to release chondrocytes from the tissue. Full-thickness human cartilage collected from an amputated ankle joint of a patient (male, 45 years old) without arthritis history was digested with the same enzymes for the isolation of chondrocytes. Bovine chondrocytes were cultured in DMEM/F12/10% FBS containing antibiotics inside a humidified 37°C incubator supplied with 5% CO_2_ and 5% O_2_. At day 6 or 7, in the 1st and 2nd experiments, chondrocytes were subcultured at 1 × 10^6^ cells/well in a 6-well culture plate, while in the 3rd experiment, cells were seeded at 3 × 10^6^ cells per well. After 2 or 3 days, those passage 1 (P1) cells were subjected to serum starvation 24 hrs prior to being challenged with factors shown in Table 1. Human chondrocytes were cultured in DMEM/MEM*α*/F12/10% FBS supplemented with 100 U/L insulin, 25 mg/L ascorbate, 276 ug/L hydrocortisone, and antibiotics. Cells at passage 2 (P2) were seeded in 6-well plates at 3 × 10^6^ per well and cultured for 2 days prior to serum starvation. After 24 hrs, serum-deprived P2 cells were treated with factors shown in Table 1.

### 2.2. Treatments with Defined DAMPs and/or Other Proinflammation Mediators

Immediately before the treatments, bovine or human chondrocyte cultures were replenished with fresh serum-free media. In the 1st and 2nd experiments, bovine chondrocytes were treated with synthetic MTDs including 1 or 10 nM N-formyl-met-leu-phe (fMLF) (Tocris Bioscience, Bristol, UK), 10 *μ*g/mL CpG DNA (a 22-mer oligonucleotides containing CpG motifs), and 10 *μ*g/mL CpG DNA negative control (InvivoGen, San Diego, CA). As a vehicle control to fMLF, DMSO (Sigma-Aldrich, St. Louis, MO) with a vol. equal to 10 nM fMLF was applied to designated cultures. The rest of the cultures were treated with purified bovine HMGB1 (Chondrex, Redmond, WA) at 10 ng/mL alone or in the presence of rhIL-1*β* (R&D Systems, Minneapolis, MN). In the 3rd bovine experiments and the experiments with human chondrocytes, two more positive controls, including 100 ng/mL rbTNF-*α* (R&D Systems, Minneapolis, MN) and 300 ng/mL 29 kDa fibronectin fragment (Fn-f) (a generous gift from the late Professor Gene A. Homandberg), were added to the treatments. A recombinant human HMGB1 (R&D Systems, Minneapolis, MN) replaced the purified bovine HMGB1 used in the 1st and 2nd experiments. To verify the findings in bovine chondrocyte cultures, same DAMPs were applied to human chondrocytes. Various treatments along with downstream effectors examined in our study and key findings are summarized in Table 1.

### 2.3. Treatments with Undefined DAMP Mixture Released from Bluntly Impacted Cartilage

In order to determine whether a mixture of undefined DAMPs released from mechanically injured cartilage was able to evoke MMP-3 secretion from chondrocytes, culture media containing substance diffused from mechanically impacted cartilage at 24 hr postinjury were collected and then applied to chondrocyte monolayer cultures and the expression of MMP-3 was examined after 24 hrs of incubation. Two levels of focal damage in cartilage were created by dropping a 2 kg of weight from a height of 7 cm or 14 cm onto an indenter resting on the surface of bovine cartilage of an osteochondral explant (2.0–2.5 cm in width × 2.0–2.5 cm in length × 0.5–1.0 cm in depth) aseptically sawed from bovine lateral tibial plateau. This single blunt impact on cartilage resulted in the death of 60% of superficial zone chondrocytes [10], activation of two MAP kinases [26], and generation of biologically active Fn-fs [27] in just 24 hrs postimpact. Passage 1 bovine chondrocytes from full-thickness tibial plateau cartilage were seeded at 0.3 × 10^6^ cells/cm^2^ and cultured in DMEM/F12/10% FBS for 3-4 days. After 24 hrs of serum deprivation, cells were subjected either to fresh serum-free media containing defined DAMPs or to cartilage-conditioned media for 1 day.

### 2.4. Examination of Expression of MMPs in Culture Medium and Expression of iNOS in Cell Lysates with Western Blotting

After 24 hrs of stimulation with DAMPs, culture media were dialyzed and concentrated for examination of MMP-1, MMP-3, MMP-13, ADAMTS-5, and ADAM-8. Meanwhile, chondrocytes were lyzed with lysis buffer containing protease and phosphatase inhibitors and supernatants were probed for the expression of iNOS. Prepared medium samples at the same vol. or cell lysates containing same amount of total proteins were resolved by 10% SDS-PAGE. After electrophoresis, proteins were blotted onto nitrocellulose membrane and then the membrane was blocked with 3% BSA/TBS. After blocking, blots were incubated with 1% BSA/TBST containing 1 : 2000 diluted anti-MMP-1 antibody (Abcam®, Cambridge, MA), or 1 : 3000 diluted anti-MMP-3 antibody (BIOMOL International, Kelayres, PA; Abcam, Cambridge, MA), or 1 : 1000 diluted anti-MMP-13 antibody (Abcam, Cambridge, MA), or 1 : 1000 diluted anti-ADAM-8 antibody (Sigma-Aldrich, St. Louis, MO), or 1 : 1000 diluted anti-ADAMTS-5 antibody (Abcam, Cambridge, MA), or 1 : 5000 diluted anti-iNOS antibody (BD Transduction Laboratories, Sparks, MD) and then incubated with goat anti-rabbit IgG antibody linked with HRP (Sigma-Aldrich, St. Louis, MO). Chemiluminescent signals were revealed with SuperSignal West Dura Chemiluminescent Substrate (Thermo Fisher Scientific Inc., Rockford, IL) and captured with Kodak BioMax MR film (Sigma-Aldrich, St. Louis, MO) or Blue Classic Autoradiography film (RPI, Mount Prospect, IL).

### 2.5. Quantification and Comparison of Chondrolytic Effect of DAMPs and/or Other Proinflammatory Mediators on Chondrocytes

The mean brightness of each pixel and the number of pixels of each band in an inverted blot were quantified with Adobe Photoshop C3. Next, the absolute intensity of each band was computed by multiplying the mean brightness by the number of pixels. The relative intensity of a band was obtained by dividing the absolute intensity of the target band by that of a control band.

## 3. Results

### 3.1. In the Initial Two Experiments with Bovine Chondrocytes, HMGB1 Synergized with IL-1*β* on Upregulating Metalloproteinase Production While MTDs or HMGB1 Alone Showed Little Effect

**Figure 1:**
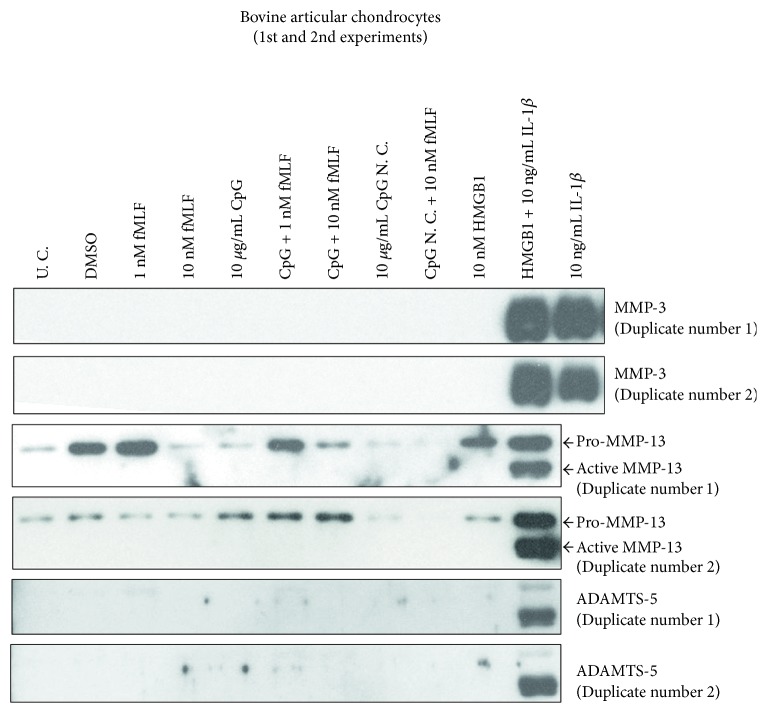
In the initial two experiments with bovine chondrocytes, HMGB1 synergized with IL-1*β* on upregulating metalloproteinase production while MTDs (fMLF and CpG DNA) or HMGB1 alone showed little effect. Cells were harvested from cartilage in bovine stifle joints and cultured for 1 week. Passage 1 cells were treated with MTDs, HMGB1, and/or IL-1*β*. After 24 hrs, culture medium was collected, dialyzed, and concentrated. The expression of MMPs in each medium sample was determined with Western blotting. U. C. = untreated control; CpG = CpG-rich DNA; CpG N. C. = CpG-rich DNA negative control.

In the first two sets of experiments with bovine chondrocytes, noticeably more MMP-3 secretion was detected in cultures treated with IL-1*β* in the presence of HMGB1 than with IL-1*β* alone (Figure 1, lane 11 versus lane 12). However, HMGB1 alone did not upregulate the expression of MMP-3, MMP-13, or ADAMTS-5 (Figure 1, lane 10) although it did upregulate the expression of pro-MMP-13 moderately. Similar to HMGB1, neither individual MTDs nor combined ones stimulated bovine chondrocytes to secrete detectable MMP-3, active MMP-13, or ADAMTS-5 (Figure 1, lanes 3–7). As a negative control for CpG DNA, a 22-mer GpC DNA alone or with 10 nM fMLF did not evoke detectable production of any of these three metalloproteinases (Figure 1, lanes 8-9).

### 3.2. The Synergism between HMGB1 and IL-1*β* Was Replicable; Such Synergism Was Also Observed between HMGB1 or MTDs and TNF-*α* but to a Lesser Extent; HMGB1 or MTDs Alone or in Combination Did Not Evoke Secretion of MMPs from Bovine Chondrocytes, Which Was Consistent with What Was Observed in Previous Two Experiments

**Figure 2:**
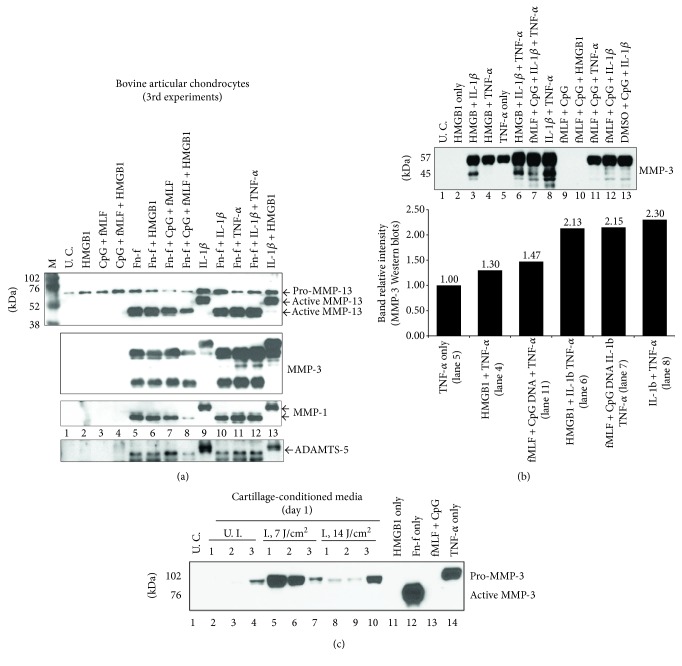
The synergism between HMGB1 and IL-1*β* was replicable in the 3rd experiment with bovine chondrocytes. Such synergism was also observed between DAMPs and TNF-*α* but to a lesser extent. However, DAMPs were unable to synergize with Fn-f on the upregulation of MMPs while the synergism between cytokines and Fn-f was observed. Moreover, DAMPs alone or in combination did not evoke secretion of MMPs from bovine chondrocytes, which was consistent with what was observed in previous two experiments. Passage 1 bovine chondrocytes were treated with DAMPs with or without Fn-f (a), with or without proinflammatory cytokines (TNF-*α* or IL-1*β*) (b) for 24 hrs. Culture medium was then examined for MMP expression with Western blotting. In addition, some cells were insulted with culture medium containing soluble substances released from injured cartilage which was bluntly impacted at 7 or 14 J/cm^2^ and cultured for 1 day. After 24 hrs of incubation, culture medium was resolved by SDS-PAGE side by side with medium samples from cultures treated with HMGB1, or MTDs, or Fn-f, or TNF-*α*. Expression of MMP-3 was then examined with immunoblotting (c). U. C. = untreated control; U. I. = unimpacted control; I. = impacted.

In the 3rd experiment with bovine chondrocytes, the synergism between HMGB1 and IL-1*β* on MMP-3 upregulation was reproduced (Figure 2(a), lane 9 versus lane 13) although this synergistic effect was not observed on the upregulation of MMP-1, MMP-13, or ADAMTS-5 (Figure 2(a), lane 13 versus lane 9). Furthermore, TNF-*α*-induced MMP-3 upregulation was as well enhanced by HMGB1 but to a lesser extent (Figure 2(b), lane 4 versus lane 5). Interestingly, MTDs exhibited similar synergistic effect to HMGB1 on TNF-*α*-induced MMP-3 upregulation (Figure 2(b), lane 11 versus lane 5).

However, the effect of the cytokine duo, IL-1*β* and TNF-*α*, on MMP-3 induction was not further enhanced by either HMGB1 or MTDs (Figure 2(b), lane 8 versus lane 6 or 7). Moreover, MTDs alone or HMGB1 did not stimulate any detectable secretion of active MMP-13, MMP-3, MMP-1, or ADAMTS-5 (Figure 2(a), lanes 2 and 3; Figure 2(b), lanes 2 and 9). This was consistent with the observations we made in the previous two experiments. Nonetheless, the combination of MTDs and HMGB1 upregulated more expression of pro-MMP-13 than did either of the reagents alone (Figure 2(a), lane 4 versus lane 2 or 3). In addition, the replacement of fMLF in MTDs with DMSO did not remarkably affect the upregulation of MMP-3 induced by MTDs and IL-1*β* (Figure 2(b), lane 13 versus lane 12).

### 3.3. DAMPs, HMGB1, or MTDs Were Unable to Synergize with Fn-f on the Upregulation of Metalloproteinases While the Synergism between Cytokines and Fn-f Was Observed

Unlike the synergism exhibited on cytokines, HMGB1 could not enhance Fn-f-induced MMP-3 upregulation (Figure 2(a), lane 6 versus lane 5). Moreover, neither MTDs alone nor in combination with HMGB1 strengthened Fn-f induced upregulation of MMP-1, or MMP-13, or ADAMTS-5 (Figure 2(a), lane 7 or 8 versus lane 5). However, the cytokines synergized with Fn-f on upregulating MMP-3 expression (Figure 2(a), lanes 10–12 versus lane 5).

### 3.4. Soluble Substances Released from Bluntly Impacted Cartilage Stimulated Bovine Chondrocytes to Upregulate Secretion of MMP-3; the Effect Might Attribute to Fn-f or Proinflammatory Cytokines rather than to HMGB1 or MTDs

Soluble substances, including DAMPs, released from traumatized cartilage within 24 hrs of injury markedly induced MMP-3 expression in bovine monolayer chondrocytes while substances released from uninjured cartilage exhibited little effect on MMP-3 induction (Figure 2(c), lanes 5–7 or 8–10 versus lanes 2–4). Between two types of injured cartilage, the one being impacted with a lesser energy density (7 J/cm^2^) released more active DAMPs than did the one being injured with a higher energy density (14 J/cm^2^) since the amount of MMP-3 averaged from 3 individual experiments was apparently more in the former than in the latter (Figure 2(c), lanes 5–7 versus lanes 8–10). However, those undefined DAMPs induced less MMP-3 than did Fn-f or TNF-*α* alone (Figure 2(c), lanes 5–7, 8–10 versus lane 12 or 14). The release of MMP-3 was not detected in untreated control (Figure 2(c), lane 1) or cells treated with either 10 nM HMGB1 (Figure 2(c), lane 11) or MTDs composed of 10 nM fMLF and 10 *μ*g/mL CpG DNA (Figure 2(c), lane 13).

### 3.5. Observations Made in Bovine Chondrocytes Were Further Verified in the Same Type of Cells in Humans; Moreover, Moderate Synergism between HMGB1 and Fn-f Was Observed on Upregulation of MMPs; Even Stronger Synergism Was Observed between IL-1*β* and Fn-f

**Figure 3:**
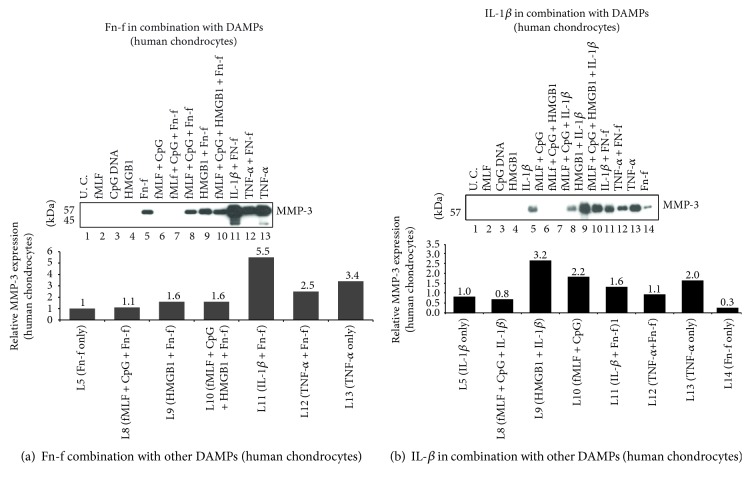
Observations made in bovine chondrocytes were further verified in the same type of cells in humans. Articular chondrocytes were isolated from the ankle cartilage of an amputated patient. Passage 2 cells were treated with DAMPs and/or Fn-f (a) or IL-1*β* (b) for 24 hrs. Medium samples were analyzed for MMP-3 expression with Western blotting. Relative intensity of protein bands on each blot was measured and plotted, respectively.

**Figure 4:**
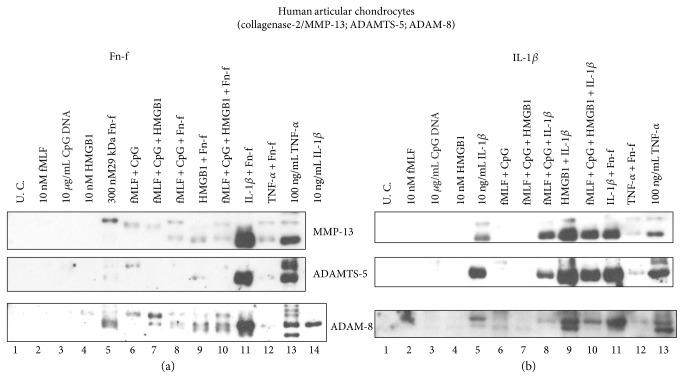
Observations made in bovine chondrocytes were further verified in the same type of cells in humans. Articular chondrocytes were isolated from ankle cartilage of an amputated patient. Passage 2 cells were treated with DAMPs and/or Fn-f (a) or IL-1*β* (b) for 24 hrs. Medium samples were analyzed for expression of MMP-13 and ADAMTS-5 with Western blotting. Furthermore, the induction pattern of ADAM-8, a newly discovered fibronectinase, was examined with the same technique.

Firstly, neither MTDs nor HMGB1 stimulated any detectable MMP-3, MMP-13, or ADAMTS-5 (lanes 2–4 and lane 6 in Figures 3(a) and 3(b) and Figure 4, respectively). None of above metalloproteinases was markedly upregulated even when those two types of DAMPs were combined (lane 7 in Figures 3(a), 3(b), and 4). Secondly, IL-1*β*-induced MMP-3 upregulation was increased by HMGB1 by 3.2-fold while Fn-f-induced MMP-3 secretion was only enhanced by 1.6-fold (Figures 3(a) and 3(b), lane 9 versus lane 1). Similar pattern was observed in the expression of MMP-13 and ADAMTS-5 (Figure 4, lane 9 versus lane 5). Moreover, compared to HMGB1, IL-1*β* was a stronger synergistic factor to Fn-f (Figure 3(a), Figure 4(a): lane 11 versus lane 5, lane 9 versus lane 5). For instance, IL-1*β* enhanced Fn-f-induced MMP-3 secretion by 5.5-fold while HMGB1 did only by 1.6-fold. (Figure 3(a), lane 11 versus lane 5, lane 9 versus lane 5). By contrast, HMGB-1 was a stronger synergistic factor to IL-1 *β* than to Fn-f (Figure 3(b), Figure 4(b): lane 9 versus lane 5, lane 11 versus lane 5). For example, in terms of MMP-3 induction, the effect of IL-1*β* was enhanced by 3.2-fold by HMGB1 while only by 1.6-fold by Fn-f (Figure 3(b), lane 9 versus lane 5, lane 11 versus lane 5). Nonetheless, this synergism between HMGB1 and Fn-f or IL-1*β* was not further strengthened by MTDs (Figures 3(a) and 3(b) and Figure 4, lane 10 versus lane 9). Interestingly, Fn-f did not synergize with TNF-*α* on the upregulation of MMP-3, MMP-13, or ADAMTS-5 (Figures 3(a) and 3(b) and Figure 4, lane 12 versus lane 13).

### 3.6. In Human Chondrocyte Cultures, the Induction Pattern of ADAM-8 Protein Expression by Tested Inflammation Mediators Was Similar to That of Other MMPs Examined in This Study

The effect of MTDs or HMGB1 on the upregulation of a newly discovered fibronectinase, ADAM-8, was also investigated in human articular chondrocytes. Similar to other metalloproteinases, the expression of ADAM-8 was only stimulated by Fn-f, or IL-1*β*, or TNF-*α*, or combinations containing any of those three agents (lanes 5, 8–14 in the bottom left blot of Figure 4; lanes 5, 8–13 in the bottom right blot of Figure 4). Furthermore, the combination of IL-1*β* and Fn-f stimulated more ADAM-8 production than either of the agents alone (lane 11 versus lane 5 or 14 in the bottom left blot in Figure 4) while TNF-*α* acted in an opposite way (lane 12 versus lane 5 or 13 in the bottom left blot in Figure 4). HMGB1 synergized with IL-1*β* not Fn-f on ADAM-8 upregulation (bottom blots in Figure 4, lane 9 versus lane 5) while MTDs weakened the effect of Fn-f or IL-1*β* on ADAM-8 induction (bottom blots in Figure 4, lane 8 versus lane 5). Neither MTDs nor HMGB1 alone or in combination could evoke any detectable ADAM-8 production (bottom blots in Figure 4, lanes 2–4, 6-7).

### 3.7. Protein Expression of a Nonmetalloproteinase Inflammation Downstream Effector, iNOS, Was Only Induced by IL-1*β* or TNF-*α* Not by HMGB1 or MTDs; However, HMGB1 or MTDs Did Moderately Synergize with IL-1*β* on iNOS Induction

**Figure 5:**
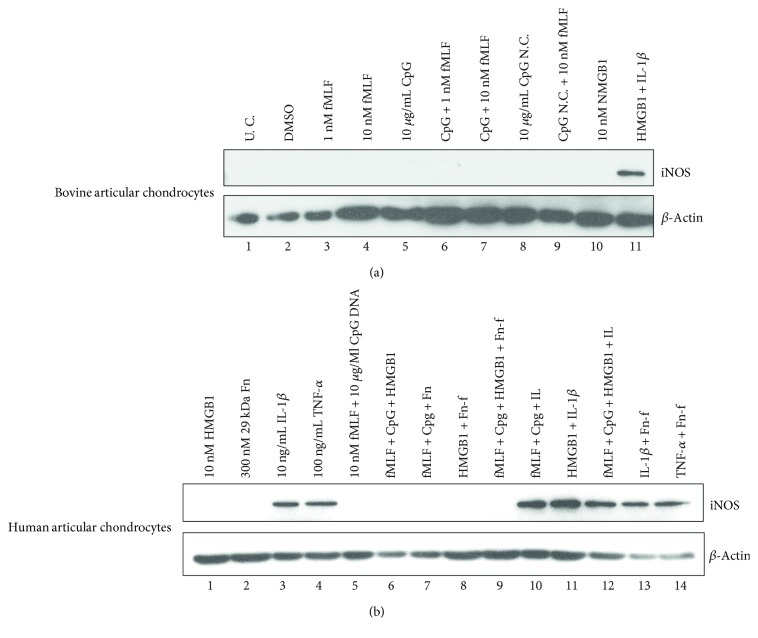
Protein expression of iNOS, an inflammation-pathway-downstream effector, was only induced by IL-1*β* or TNF-*α* not by DAMPs. Bovine (a) or human (b) articular chondrocytes were treated with DAMPs or cytokines or Fn-f or in combination for 24 hrs. Cells were then lyzed, and the lysates were examined for expression of iNOS with Western blotting.

In addition to metalloproteinases, the effect of MTDs or HMGB1 on the induction of a cytosolic inflammation mediator, iNOS, was examined. In bovine articular chondrocytes, the expression of iNOS was only detected in cultures treated with the combination of IL-1*β* and HMGB1 (Figure 5(a), lane 11). HMGB1 at a low dose (1 nM) or a high dose (10 nM) could not stimulate any detectable iNOS expression. Same observation was made in the cultures treated with either individual or complete MTDs (Figure 5(a), lanes 10, 3–7). These results were further validated in human articular chondrocytes. However, MTDs or HMGB1 did moderately enhance the effect of IL-1*β* on iNOS induction while neither agents synergized with Fn-f (Figure 5(b), lanes 10–12 versus lane 3; lanes 7-8 versus lane 2). Another proinflammatory cytokine, TNF-*α*, also induced iNOS expression. However, this action was not strengthened by the addition of Fn-f (Figure 5(b), lane 4 versus lane 14).

## 4. Discussion

Our study provided ample evidence suggesting an indirect role of MTDs and HMGB1 in chondrocyte-mediated cartilage degeneration occurred in PTOA. Unlike other proinflammatory mediators tested in this study, including IL-1*β*, TNF-*α*, and Fn-f, MTDs or HMGB1 could not upregulate cartilage matrix-degrading MMPs or stimulate the expression of iNOS. On the other hand, we did observe that HMGB1 was a strong synergistic factor to IL-1*β* rather than to TNF-*α* or Fn-f in terms of upregulating cartilage-damaging MMPs or iNOS while MTDs was a relatively weak synergic factor to TNF-*α*. To our knowledge, this is the first study examining whether DAMPs originated from ruptured mitochondria (MTDs) or nuclei (HMGB1) could induce chondrolytic response in articular chondrocytes and whether synergism existed between those types of DAMPs and proinflammatory cytokines or Fn-f.

It has been shown that MTDs composed mainly of mitochondrial DNA (mtDNA; CpG-rich DNA) and N-formyl peptides could stimulate immune cells to produce inflammation mediators. In a study by Zhang et al., mtDNA or fMLF alone could upregulate MMP-8 expression by PMNs. The combination of 10 *μ*g/mL CpG DNA and 1.0 nM fMLF was found to be effective in activating PMNs to secrete IL-8 [21]. Both MMP-8 and IL-8 facilitate migration of PMNs into bystander organs over the course of inflammatory response. Moreover, intra-articular injection of mtDNA could induce monocytes in mouse synovium to secrete TNF-*α*, which eventually led to rheumatoid arthritic changes in injected joints [28].

However, unlike PMNs or monocytes, chondrocytes did not respond to MTDs either in the form of single agent or in combinations that were tested in our study. CpG DNA at 10 *μ*g/mL, an effective dose for PMN activation, could not upregulate any MMPs or iNOS in bovine or human chondrocytes after 24 hr incubation. Same lack of response was observed when chondrocytes were challenged with combinations of CpG DNA and fMLF that showed effectiveness in upregulating MMP-8 in PMNs. Nonetheless, this unresponsiveness only informed us that MTDs might not cause direct chondrolysis but they may still play a crucial role in injury-induced cartilage degradation since data have shown that MTDs could stimulate monocytes, inside synovium or infiltrated after injuries, to produce significant amount of inflammatory cytokines, such as TNF-*α* [28]. Those cytokines are capable of upregulating MMPs in chondrocytes [23]. Interestingly, we observed moderate synergism between MTDs and TNF-*α* on upregulation of MMP-3 in bovine chondrocytes. This implicated that MTDs might induce and promote chondrolysis through the action of TNF-*α*.

HMGB1 as a nuclear nonhistone DNA-binding protein not only regulates gene transcription but also acts as a cytokine to amplify inflammatory response based on recent reports [29]. Andersson and colleagues showed that HMGB1 significantly stimulated peripheral blood monocytes to release proinflammatory cytokines including TNF-*α*, IL-1*α*, IL-1*β*, IL-6, IL-8, macrophage inflammatory protein-1*α* (MIP-1*α*), and MIP-1*β*. Furthermore, they demonstrated that this proinflammatory role of HMGB1 was cell type specific since lymphocytes could not be stimulated by the same doses of HMGB1 that were effective for monocytes [30]. In terms of the effect of HMGB1 on chondrocytes, studies showed that HMGB1 could act as a cytokine to upregulate protein expression of MMP-3, MMP-13, or iNOS [31–33]. However, in our study we only observed that HMGB1 moderately upregulated pro-MMP-13 in bovine chondrocytes. The reasons could be as follows: (1) we used normal bovine or human chondrocytes not osteoarthritic human chondrocytes or immature mouse chondrocytes examined in the studies mentioned above and (2) the dose of HMGB1 tested in our study was much lower than that tested in those studies (10 nM or 0.3 *μ*g/mL in our study versus 2.5 or 5 or 10 *μ*g/mL in other studies). In fact, our results were consistent with what Ley et al. reported in their study comparing the effect of HMGB1, IL-1, and IL-6 on cartilage matrix metabolism. They described that HMGB1 at 1.0 *μ*g/mL could not stimulate equine chondrocytes to produce MMP-13 protein [34].

Another reason for cells not responding to HMGB1 stimulation observed in our study could be the redox status change of the protein. Studies showed that HMGB1 lost cytokine activities when the protein was oxidized on thiol groups of three Cys residues at positions 23, 45, and 106 by reactive oxygen or nitrogen species (ROS or RNS) [35, 36]. This could explain why HMGB1 in bluntly impacted cartilage-conditioned media might not be one of the inflammatory mediators that upregulated MMP-3 in chondrocyte cultures examined in our study since blunt impact on cartilage created strong oxidative milieu [37] which could terminally oxidize HMGB1 that was mainly released from necrotic chondrocytes within 24 hrs of the injury [10].

Although HMGB1 could not stimulate chondrocytes to synthesize detectable MMPs or iNOS in our study, this nuclear DAMP synergized with IL-1*β* to amplify the inflammatory response. Similar results were reported by García-Arnandis et al. who made observations about HMGB1 potentiating the proinflammatory effects of IL-1*β* on synoviocytes. In the absence of IL-1*β*, HMGB1 either at a low dose (15 ng/mL) or a high dose (25 ng/mL; ~1 nM) stimulated barely detectable amount of MMP-1, MMP-3, and MMP-13 at mRNA and protein levels. However, in the presence of 10 ng/mL IL-1*β*, either dose of HMGB1 induced significantly more expression of MMP-1 and MMP-3 than did IL-1*β* work alone [38].

In our study, the synergistic effect between HMGB1 and IL-1*β* on MMP-3 upregulation was detected in both bovine and human chondrocytes while the synergism on the expression of MMP-13 or ADAMTS-5 was observed only in human cells. This could be attributed to the fact that the recombinant IL-1*β* protein used in our study was human origin (R&D Systems, Cat^#^201-LB) and might cause less strength of stimulation to bovine cells than to human cells. MMP-3 as an activator of other MMPs [39] needs to be induced in a much more sensitive manner and may not be affected by the origin of species of IL-1*β* protein.

Since HMGB1 may interact with membrane receptors in chondrocytes [40–43], such as TLRs, RAGE, or CXCRs, to elicit and prolong proinflammatory effect, the involvement of those receptors in trauma-induced chondrolysis will be investigated in our future studies. The investigation will help us to understand the synergistic mechanism between HMGB1 and Fn-f that was only observed in human chondrocytes since a recent study showed that Fn-f signaled through TLR-2 to induce MMP upregulation in human chondrocytes [44].

Our data indicated that contents leaked from ruptured mitochondria or nuclei in necrotic cells following joint injuries might not directly evoke inflammation cascades in chondrocytes. Their target cells might be synoviocytes which could respond to those MTDs or HMGB1 by secreting inflammatory cytokines that could in turn stimulate chondrocytes to produce matrix-degrading MMPs and other mediators involved in chondrolysis. Furthermore, HMGB1 as a NuD could markedly amplify the proinflammatory effect of IL-1*β* on synoviocytes and chondrocytes. Therefore, strategies that may prevent the interactions between MTDs or HMGB1 and synoviocytes or block IL-1*β* signaling ought to attenuate mechanical-injury-induced cartilage degeneration. Future studies will employ ex vivo and in vivo experimental models to examine those speculations.

## Figures and Tables

**Table 1:** Summary of various types of treatment involving DAPMs examined in the study.

Treatments (DAMPs/Fn-f/proinflammatory cytokines)			Doses		Downstream effector tested	Summary of results	Figure number
Individual or undefined	Synthetic	fMLF	1 nM	10 nM	MMP-3, MMP-13, ADAMTS-5, ADAM-8, iNOS	(1) Moderate upregulation of pro-MMP-13 only induced in bovine cells (2) No detectable upregulation of active form of MMP-13 or of other tested effectors	1, 3(a), 3(b), 4, 5(a)
		CpG DNA	10 *μ*g/mL				
		rh HMGB1	10 nM		MMP-1, MMP-3, MMP-13, ADAMTS-5, ADAM-8, iNOS		1, 2(a), 2(b), 2(c), 3(a), 3(b), 4, 5(a), 5(b)
		rhIL-1*β*	10 ng/mL		MMP-1, MMP-3, MMP-13, ADAMTS-5, ADAM-8, iNOS	Strong upregulation of tested effectors	1, 2(a), 3(b), 4(b), 5(b)
		rbTNF-*α*	100 ng/mL		MMP-3, MMP-13, ADAMTS-5, ADAM-8, iNOS		2(b), 3(a), 3(b), 4(b), 5(b)

Individual or undefined	Nonsynthetic	N-terminal 29 kDa Fn-f	300 nM		MMP-1, MMP-3, MMP-13, ADAMTS-5, ADAM-8, iNOS	Strong upregulation of tested effectors except iNOS	2(a), 3(a), 3(b), 4(a), 5(b)
		Injured cartilage-conditioned media	Day 1 postinjury		MMP-3	Strong upregulation of MMP-3	2(c)

Combined	Paired	MTDs	10 *μ*g/mL CpG DNA + 1 nM fMLF	10 *μ*g/mL CpG DNA + 10 nM fMLF	MMP-1, MMP-3, MMP-13, ADAMTS-5, ADAM-8, iNOS	No detectable upregulation of tested effectors	1, 2(a), 2(b), 2(c), 3(a), 3(b), 4, 5(a), 5(b)
		Fn-f with individual DAMPs or proinflammatory cytokines	300 nM Fn-f + 10 nM HMGB1		MMP-1, MMP-3, MMP-13, ADAMTS-5, ADAM-8, iNOS	(1) Strong upregulation of tested effectors (2) Marked synergism between Fn-f and cytokines observed in MMP-3 upregulation (3) In human cells, moderate synergism observed between HMGB1 and Fn-f on upregulating MMP-3, MMP-13, and ADAMTS-5	2(a), 3(a), 4, 5(b)
			300 nM Fn-f + 10 ng/mL IL-1*β*				
			300 nM Fn-f + 100 ng/mL TNF-*α*				
		Proinflammatory cytokines or with individual DAMPs	10 ng/mL IL-1*β* + 100 ng/mL TNF-*α*		MMP-3	(1) IL-1*β* and TNF-*α* induced the most MMP-3 protein expression (2) Moderate synergism shown between TNF-a and HMGB1	2(b)
			100 ng/mL TNF-*α* + 10 nM HMGB1				
			10 ng/mL IL-1*β* + 10 nM HMGB1		MMP-1, MMP-3, MMP-13, ADAMTS-5, ADAM-8, iNOS	Strong synergism shown between HMGB1 and IL-1*β* on upregulating MMP-3, MMP-13, ADAMTS-5, ADAM-8, or iNOS	2(a), 2(b), 3(b), 4(b), 5(b)
Combined	Triple	MTDs with Fn-f or proinflammatory cytokines	10 *μ*g/mL CpG DNA + 10 nM fMLF + 10 nM HMGB1		MMP-1, MMP-3, MMP-13, ADAMTS-5, ADAM-8, iNOS	(1) In bovine cells, moderate upregulation of pro-MMP-13 induced weaker effect in human cells (2) No detectable upregulation of active MMP-13 or other tested effectors	2(a), 2(b), 3(a), 3(b), 4, 5(b)
			10 *μ*g/mL CpG DNA + 10 nM fMLF + 300 nM fMLF		MMP-1, MMP-3, MMP-13, ADAMTS-5, ADAM-8, iNOS	(1) Strong upregulation of tested effectors expect iNOS (2) Synergism not observed	2(a), 3(a), 4(a), 5(b)
			10 *μ*g/mL CpG DNA + 10 nM fMLF + 10 ng/mL IL-1*β*		MMP-1, MMP-13, ADAMTS-5, ADAM-8, iNOS	(1) Strong upregulation of tested effectors (2) Moderate synergism observed on upregulating MMP-13 or iNOS	2(b), 3(a), 4(b), 5(b)
			10 *μ*g/mL CpG DNA + 10 nM fMLF + 100 ng/mL TNF-*α*		MMP-3	HMGB1 and IL-1*β* showed much stronger synergism with TNF-*α* than MTDs did	2(b)
		Proinflammatory cytokines with HMGB1 or Fn-f	10 nM HMGB1 + 10 ng/mL IL-*β* + 100 ng/mL TNF-*α*				
			300 nM Fn-f + 10 ng/mL IL-*β* + 100 ng/mL TNF-*α*		MMP-1, MMP-3, MMP-13, ADAMTS-5	Strong upregulation of tested effectors	2(a)

Combined	Quaternary	DAMPs with Fn-f or proinflammatory cytokines	10 *μ*g/mL CpG DNA + 10 nM fMLF + 10 nM HMGB1 + 300 nM Fn-f		MMP-1, MMP-3, MMP-13, ADAMTS-5, ADAM-8, iNOS	Strong upregulation of tested effectors except iNOS	2(a), 3(a), 4(a), 5(b)
			10 *μ*g/mL CpG DNA + 10 nM fMLF + 10 nM HMGB1 + 10 ng/mL IL-1*β*		MMP-3, MMP-13, ADAMTS-5, ADAM-8, iNOS	(1) Strong upregulation of tested effectors (2) Marked synergism observed on upregulating MMP-3, MMP-13, or iNOS	3(b), 4(b), 5(b)
			10 *μ*g/mL CpG DNA + 10 nM fMLF + 10 ng/mL IL-1*β* + 100 ng/mL TNF-*α*		MMP-3	Strong upregulation of MMP-3 observed but no synergism between MTDs and cytokines detected	2(b)
